# Unique territorial and compartmental organization of chromosomes in the holocentric silkmoth

**DOI:** 10.21203/rs.3.rs-4732646/v1

**Published:** 2024-07-31

**Authors:** J. Gil, E. Navarrete, L.F. Rosin, N. Chowdhury, S. Abraham, G. Cornilleau, E.P. Lei, J. Mozziconacci, L.A. Mirny, H. Muller, I.A. Drinnenberg

**Affiliations:** 1 Institut Curie, PSL University, Sorbonne Université, CNRS, Nuclear Dynamics, 75005 Paris, France; 2 Department of Biology, Massachusetts Institute of Technology, Cambridge, MA 02139, USA; 3 Institute for Medical Engineering and Science, and Department of Physics, Massachusetts Institute of Technology, Cambridge, MA 02139, USA; 4 Nuclear Organization and Gene Expression Section; Laboratory of Biochemistry and Genetics, National Institute of Diabetes and Digestive and Kidney Diseases, National Institutes of Health, Bethesda, MD 20892 USA; 5 StrInG Lab, Museum National d’Histoire Naturelle, Paris, France

## Abstract

The hallmarks of chromosome organization in multicellular eukaryotes are chromosome territories (CT), chromatin compartments, and insulated domains, including topologically associated domains (TADs). Yet, most of these elements of chromosome organization are derived from analyses of a limited set of model organisms, while large eukaryotic groups, including insects, remain mostly unexplored. Here we combine Hi-C, biophysical modeling, and microscopy to characterize the 3D genome architecture of the silkmoth, *Bombyx mori*. In contrast to other eukaryotes, *B. mori* chromosomes form highly separated territories. Similar to other eukaryotes, *B. mori* chromosomes segregate into active A and inactive B compartments, yet unlike in vertebrate systems, contacts between euchromatic A regions appear to be a strong driver of compartmentalization. Remarkably, we also identify a third compartment, called secluded “S,” with a unique contact pattern. Each S region shows prominent short-range self-contacts and is remarkably devoid of contacts with the rest of the chromosome, including other S regions. Compartment S hosts a unique combination of genetic and epigenetic features, localizes towards the periphery of CTs, and shows developmental plasticity. Biophysical modeling reveals that the formation of such secluded domains requires highly localized loop extrusion within them, along with a low level of extrusion in A and B. Our Hi-C data supports predicted genome-wide and localized extrusion. Such a broad, non-uniform distribution of extruders has not been seen in other organisms. Overall, our analyses support loop extrusion in insects and highlight the evolutionary plasticity of 3D genome organization, driven by a new combination of known processes.

## Introduction

The development of high-throughput chromosome conformation capture (Hi-C) has uncovered the rich hierarchy of structures in the genome ^1–3^. At the highest level, full chromosomes occupy distinct territories within the nucleus (chromosome territories, CTs). Next, sub-chromosomal segments of like epigenetic state phase separate into active (A) and inactive (B) compartments. Finally, molecular motors belonging to the structural maintenance of chromosomes (SMC) complexes processively bridge chromatin contacts, generating structures such as Topologically Associating Domains (TADs) and fountains or jets^1,2,4–17^.

Much of this knowledge stems from in-depth studies of model organisms, such as mammals, flies, yeasts, bacteria, and plants ^4–7,17–24^. While many Hi-C datasets have been generated for non-model organisms to aid in their genome assemblies ^25–29^, studies of the underlying structures in these Hi-C maps are far less common. Nevertheless, such studies of non-model organisms continue to uncover new modes of chromosome organization ^30–32^.

While one recent study analyzed and compared genome organization among a diverse set of organisms,^3^ it excluded species with radically different linear chromosome organization, such as species with holocentric chromosomes ^3^. Unlike most eukaryotes that have a single, locally restricted (mono-)centromeric regions per chromosome, holocentric organisms have many centromeric regions distributed along the entire length of chromosomes. Analyses of the 3D spatial organization of holocentric chromosomes in interphase could lead to new organizational principles of the genome and help to deduce the role of centromeres in chromatin organization ^33,34^.

Holocentric chromosomes have evolved convergently many times across a broad range of animal and plant species ^35^. Nevertheless, the 3D genome organization of holocentric organisms remains poorly understood. A notable exception is the nematode *Caenorhabditis elegans* ^36–39^. At a larger scale, *C. elegans* chromosomes spatially segregate into a tripartite structure reflecting its linear organization ^40^ with preferential contacts between arm and center regions in *cis* and *trans* ^37^. At a smaller scale, chromatin compartmentalizes according to its epigenetic state ^39^. In addition, signatures of SMC-mediated loop extrusion have also been described in *C. elegans*. These include TAD-like structures restricted to the X chromosome ^36,38^, and targeted loading of SMCs at active enhancers generate structures termed fountains ^11,39^. While many interesting chromosome folding structures have been found in *C. elegans*, it is unclear whether any of these structures are associated with holocentricity.

Here, we provide an in-depth characterization of the 3D genome architecture of another holocentric species, the silkmoth *Bombyx mori*. We combine Hi-C with Oligopaint FISH analyses and computational modeling to determine the folding principles of *B. mori* chromosomes. Our analyses reveal a new type of chromatin compartment that results from the interplay of affinity-mediated interactions and locally concentrated loop extrusion. Our study thus demonstrates that a new combination of classical drivers of spatial genome organization can lead to new 3D landscapes.

## Results

### Bombyx mori linear genome organization

**Figure 1:**
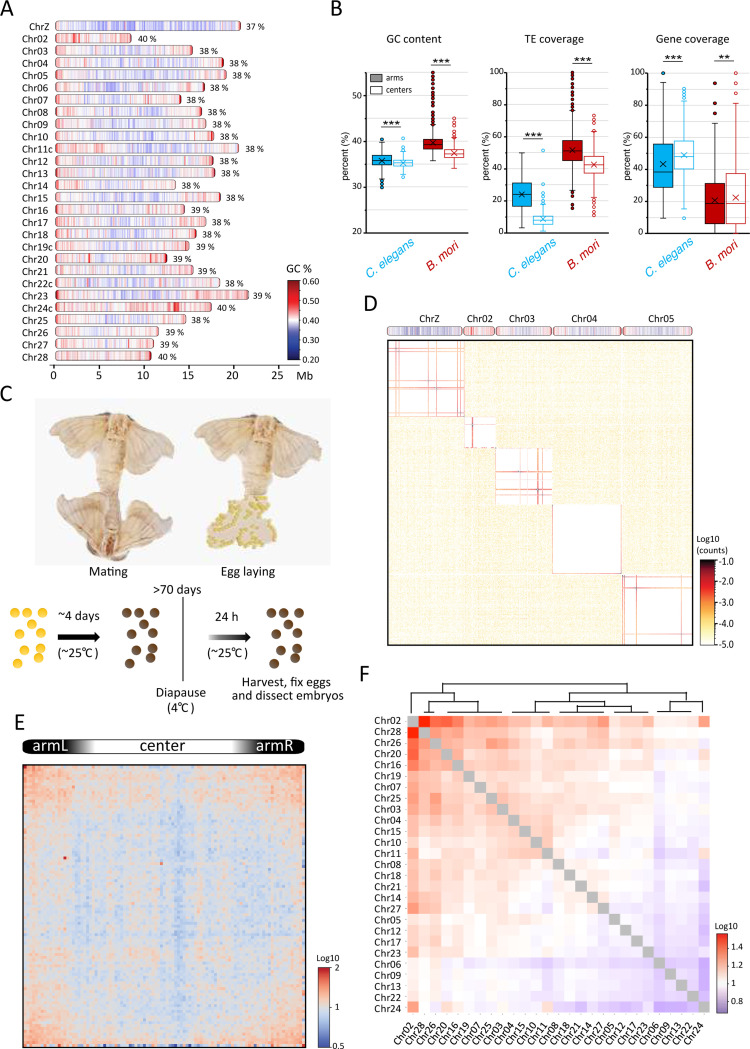
Hi-C of *Bombyx mori* embryos reveals highly distinct chromosome territories. (**A**) Schematics of *B. mori* chromosomes drawn to approximate scale indicated in Mb below. For each chromosome, total GC content in percentage is noted, and local GC content per 100 kb windows is indicated as a color scale from blue (<20%) to red (>60%). A suffix “c” added to a chromosome’s name indicates assembly corrections were made (Figure S9). (**B**) Box plots showing the distribution of GC content, TE coverage, and gene density per 100 kb window in center and arm regions of autosomes in *C. elegans* and *B. mori*. Statistical significance was tested using a Mann-Whitney U-test, **: P-value < 0.05, ***: P-value < 0.005. Box region corresponds to data between the first and third quartile. Lines indicate medians of respective distributions, while crosses correspond to their means. Whiskers extend to the lowest and highest data points, excluding outliers, which are shown as dots. (**C**) Sample generation for Hi-C and ChIP-seq. (**D**) Contact map at 80 kb bin resolution of five selected *B. mori* chromosomes (*on top*). (**E**) Average inter-chromosomal (*trans*) observed contacts versus expected matrix for all scaled *B. mori* chromosomes, computed at 40 kb bin resolution. (**F**) Heatmap illustrating observed-over-expected inter-chromosomal contact frequencies as a divergent color scale from blue to red. Chromosomes have been clustered and ordered to reflect similar contact patterns.

The *B. mori* genome encompasses 27 autosomes and the Z sex chromosome (Chr01), and the W sex chromosome, which vary in length from ~8 to 22 Mb. Genome-wide analysis of GC content revealed a tripartite organization of *B. mori* chromosomes, with a central GC-poor region and two large telomere-proximal GC-rich regions (Figure 1A). This linear organization is reminiscent of observations in the nematode *C. elegans* (Figure 1B), another holocentric organism whose chromosomes are divided into centers and arms ^41^. We thus used our GC content track along *B. mori* chromosomes to define arms and center regions for each chromosome (see M&M for detail, Table S1). In *C. elegans*, chromosomal arms are enriched with repetitive DNA, while centers are enriched with genes ^41^ (Figure 1B). In *B. mori*, we found a similar organization with significant but less distinct patterns of transposable element (TE) and gene coverage (Figure 1B). The similarity in linear genome organization between these two species might reflect convergent evolution between the two holocentric lineages that evolved independently from different monocentric ancestors ^35,42^.

### Chromosomes in B. mori embryos form highly segregated territories

We generated Hi-C datasets from *B. mori* embryos at three different embryonic post-diapause time points (2, 24, and 48 hours after diapause release) and one adult stage from the p50 reference strain. For most of our analysis, we focused on the 24-hour post-diapause timepoint (PD-D2) (Figure 1C), for which we confirmed that a proportion of cells have re-entered the cell cycle and are thus no longer arrested in G2 (Figure S1) ^43^.

The PD-D2 Hi-C contact pattern across all 28 chromosomes revealed sharply demarcated contact regions for each chromosome, with very sparse inter-chromosomal contacts (Figure 1D). This is consistent with recent DNA FISH data that revealed that *B. mori* chromosomes are tightly folded and occupy distinct CTs ^44^. Despite the similarity in linear genome organization between *B. mori* and *C. elegans*, clustering between centers and arms of different chromosomes, easily seen on *C. elegans* contact maps ^36,37^, is not evident in *B. mori*. Nevertheless, drawing the average *trans* contact matrix reveals enrichments between large sub-telomeric regions, indicating some extent of telomere or arm clustering between chromosomes in *B. mori* (Figure 1E).

Although inter-chromosomal contacts are sparse, we tested whether known patterns of sub-nuclear positioning of chromosomes seen in other organisms are present in *B. mori*. In humans, small, gene-rich chromosomes have been shown to preferentially interact with each other and localize more centrally within the nucleus ^1,45–47^. A length-dependent contact preference can also be identified among *B. mori* chromosomes, with a group of small chromosomes (Chr02, 28, 26, 20, and 16) having the highest average inter-chromosomal contact frequency (Figures 1F, S2A). However, the correlation between inter-chromosomal contacts and gene density is mild (Figure S2B). This might be due to the lower variation in length and gene content among *B. mori* chromosomes compared to human chromosomes. We also noticed a positive correlation between inter-chromosomal contacts and GC content (Figure S2C), but how these factors contribute to contact preferences remains unclear.

Our data reveal conserved principles in chromosome organization in *B. mori*, such as the formation of CTs and mild length-dependent inter-chromosomal contact preferences. In contrast to other organisms, however, inter-chromosomal contacts are not correlated with gene density, and *B. mori* chromosomes make very limited inter-chromosomal contacts overall, highlighting the remarkably strong CTs in this organism.

### B. mori chromosomes are organized in three chromatin compartments

**Figure 2:**
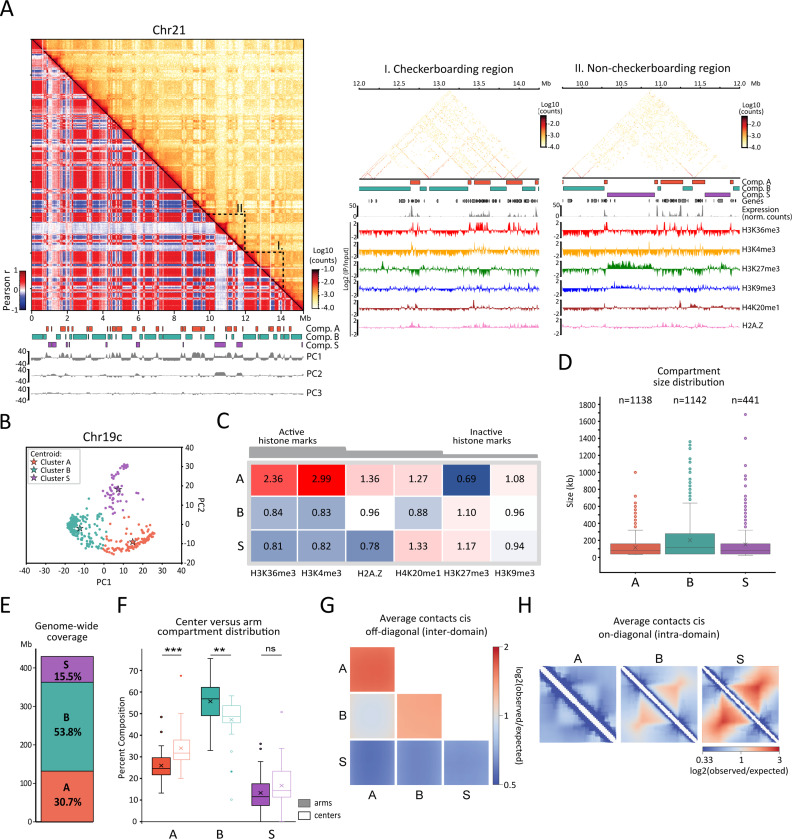
Hi-C compartmental analysis reveals three genome-wide contact patterns. (**A**) *Left*: top diagonal represents the iterative-corrected Hi-C contact map of Chr21 at 40 kb bin resolution, and bottom diagonal is the corresponding Pearson correlation matrix. Below the matrix are the gene track (black), color boxes indicating the locations of domains of compartment A (orange), B (turquoise), and S (purple) and tracks of the first three principal components (gray) computed from the correlation matrix. *Right*: close-up on regions highlighted on the left, I: checkerboarding region, II: non-checkerboarding region. Below matrices are tracks for compartments and genes as in the left as well as corresponding RNA-seq at 1 kb resolution and ChIP-seq tracks for various histone marks at 25 bp resolution. (**B**) Example of clustering along PC1 and PC2 of Pearson correlation matrix for Chr19c. Clusters called using k-means function of scikit-learn are shown in different colors. For each cluster, the centroid and the corresponding assignment to A, B, and S compartments is indicated. (**C**) Heatmap showing genome-wide enrichment of several histone marks across compartment A, B, and S. Enrichment values correspond to median of IP/input ratios at 40 kb resolution normalized to the genome-wide median for each mark. (**D**) Box plots showing the size distribution of A, B, and S domains. Boxed regions correspond to data between the first and third quartile. Lines indicate the medians of respective distributions, while crosses correspond to their means. Whiskers extend to the lowest and highest data points, excluding outliers, shown by dots. (**E**) Bar graph showing relative genomic coverage of A, B, and S. (**F**) Box plots showing coverage of compartment types A, B, and S in arms (filled boxes) or centers (transparent boxes) of *B. mori* chromosomes. Each value corresponds to the relative coverage (% in bp) of the respective compartment compared to the sizes of arms or center regions for one chromosome. Statistical significance was tested using a Mann-Whitney test; ***: P-value < 0.005. The boxed region corresponds to data between the first and third quartile. Lines indicate medians of respective distributions, while crosses correspond to their means. Whiskers extend to the lowest and highest data points, excluding outliers, shown by dots. (**G**) Average *cis* off-diagonal (inter-domain) contact versus expected plots within and between all scaled A, B, and S compartments. (**H**) Rescaled average *cis* on-diagonal (intra-domain) contact frequency compared to expected for the three compartment types.

We next explored the intra-chromosomal contact maps of *B. mori* for signatures of chromatin compartmentalization. The maps show a characteristic checkerboard pattern of alternating regions of two types, displaying enriched homotypic (self-to-self) and depleted heterotypic (self-to-other) contacts (Figure 2A, left and middle panels). This is similar to other organisms in which genomic loci spatially segregate into an active A and an inactive B compartment at the sub-chromosomal scale ^7,17–21,48^. Unexpectedly, our initial visual inspection also revealed other regions that do not conform to the checkerboard pattern. Instead, these regions engage in very frequent short-range contacts but few longer-range contacts in *cis* with any other chromosomal segments (Figure 2A, left and right panels). Thus, we aimed to identify chromosomal loci that belong to these three contact patterns. To do so, we applied an approach similar to one recently developed to detect more than two compartments ^34^. We k-means clustered the leading principal components (PCs) of the Pearson-correlated matrices, revealing groups of loci with similar contact profiles (Figures 2A, 2B, and Table S2). This strategy, for the three leading PCs at 40 kb resolution, enabled us to define three main clusters for each chromosome, which we then unified across chromosomes based on epigenetic composition (see M&M).

To further characterize the three clusters, we profiled active (H3K36me3, H3K4me3) and silent (H3K27me3, H3K9me3) histone marks by ChIP-seq in the same PD-D2 embryonic stage of *B. mori*. We also included H2A.Z, a histone variant associated with transcriptional control ^49^, that is enriched in a sub-compartment that shows an attenuated checkerboarding pattern in colon cancer cells ^34^. Additionally, we included H4K20me1, a mark associated with centromeric nucleosomes in vertebrates ^50^, as well as a variety of processes including transcriptional regulation, chromosome replication and segregation, DNA damage response, and chromosome compaction ^51,52^.

We found that one of the clusters is particularly enriched for active marks (Figures 2A, 2C, S3A, and S3B). We termed this “compartment A” as it is reminiscent of the active compartment first described in humans ^1,2^. The other two clusters are depleted of active histone marks and slightly enriched in H3K27me3, a mark associated with facultative heterochromatin ^53^. We termed the inactive cluster involved in the checkerboard pattern “compartment B” (Figures 2A, 2C, S3A, and S3B). The third cluster correlates with the regions of sparse long-range contacts noticed during our initial inspection. We named this “compartment S”, reflecting its secluded, spatially segregated behavior observed in the Hi-C maps. Like compartment A, S is enriched in H4K20me1. This enrichment, however, only applies to about one-third of all S regions, which have relatively high levels of this mark (Figures S3A and S3B). The profile of H3K9me3, a mark associated with constitutive heterochromatin, is not strongly correlated with any of the three compartments (Figures 2C and S3A). Instead, this mark appears to be distributed at low levels along the chromosomes with a slight enrichment over chromosomal arms, particularly in telomere-proximal regions similar to H3K27me3 (Figures 2A and S3B).

Domains, which we define as contiguous segments of the same compartment type, of A, B, and S are heterogeneous in length and cover different fractions of the genome (Figures 2D, 2E). Overall, A and S domains are smaller, with a median size of 80 kb, while B domains are generally larger, with a median size of 120 kb. A and B compartments cover approximately 50 and 30% of the genome, respectively, while S covers 15%. Consistent with our analyses of linear genome organization in *B. mori*, we found domains of compartment A enriched in center regions of chromosomes, while domains of compartment B are enriched in the arms (Figure 2F). Domains of compartment S are distributed throughout the genome, with no preferential clustering towards chromosomal centers or arms (Figure 2F and S3C for the distribution of S domains).

To further characterize the contact patterns of compartments A, B, and S, we calculated the average *cis*-off-diagonal (inter) contact plots for all combinations of A, B, and S domains (Figure 2G). We found that the strongest enrichment corresponds to A-A homotypic contacts, while S contacts are depleted with any of the three types to levels that are even below those between A and B. On the other hand, the average *cis* on-diagonal (intra) contact plots for each compartment demonstrate that S domains are the most compact, followed by B and A, with the latter one being in the range of the genome-wide average (Figure 2H).

We conclude that chromosomes segregate into three intra-chromosomal compartments. As is common in other organisms, we identified A and B compartments corresponding to epigenetically defined active and inactive regions of the genome. The newly identified compartment S shows a distinct pattern of contacts, unlike compartments seen in other organisms. S exhibits repressive epigenetic features with a specific enrichment of H4K20me1 and covers about one-sixth of the genome.

### Compartment S corresponds to transcribed regions of low gene density

**Figure 3:**
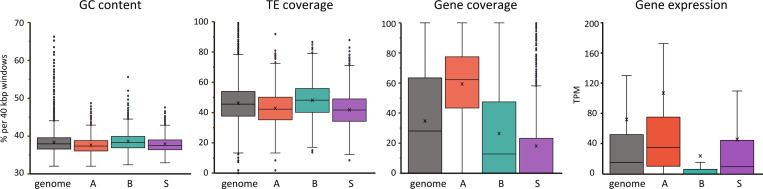
Compartment S is GC-poor, repeat-poor, and depleted in genes. Box plots showing distribution of genetic features including GC percentage, TE coverage, and gene coverage per 40 kb window of whole genome (n=11143) or within A (n=3294), B (n=5772), and S (n=1670), or per gene for gene expression (in TPM) (whole genome n=13869, in A n=7404, in B n=4500, in S n=608). Boxed regions correspond to data between the first to third quartile. Line indicates median of distribution while cross corresponds to mean. Whiskers extend to lowest and highest data points, excluding outliers, which have been removed. All distributions in each compartment for all features are significantly different from each other and from the genome-wide distribution by the Mann-Whitney (95%, two-tailed) U-test.

The distinct contact pattern of the compartment S prompted us to explore whether S is distinct from A and B with respect to genetic features (Figure 3). We found that compartment S, like compartment A, has a lower GC and TE content compared to the whole genome. Despite these similarities to A, compartment S corresponds to gene-poor regions and is even more depleted in genes than compartment B. However, unlike B, S does not appear to be a repressive compartment; the expression levels of the few genes that are located within S are in the range of the genome-wide distribution. Out of the 608 genes within S, about half (300) are expressed at the PD-D2 stage (TPM > 10) (Figure 3 and Table S3), and these genes are associated with active histone marks despite an overall depletion of these marks across all S regions (Figure 2A, right panel). Gene ontology analyses revealed genes within S are enriched in DNA-binding and transcription regulation processes (Table 1). We also specifically tested for an enrichment of homeotic and Polycomb group (PcG) response genes because one of the largest S domains in the genome corresponds to the Hox cluster, which is expressed in the PD-D2 stage (Figure S4). Using previously published datasets ^54,55^, we generated a list of 399 homeotic and PcG response genes in *B. mori* and determined their position within A, B, or S (Table S4, see M&M for details). We did not find an enrichment of homeotic or PcG response genes in compartment S but rather in A (Table S4). As a whole, our analyses show that compartment S largely encompasses gene-poor regions. Nevertheless, about half of the genes that are present in S are expressed in embryos 2 hours after diapause release and are enriched for functions related to DNA-binding and transcription regulation.

### A minimal model reveals loop extrusion is key to A, B, and S compartment organization

**Figure 4:**
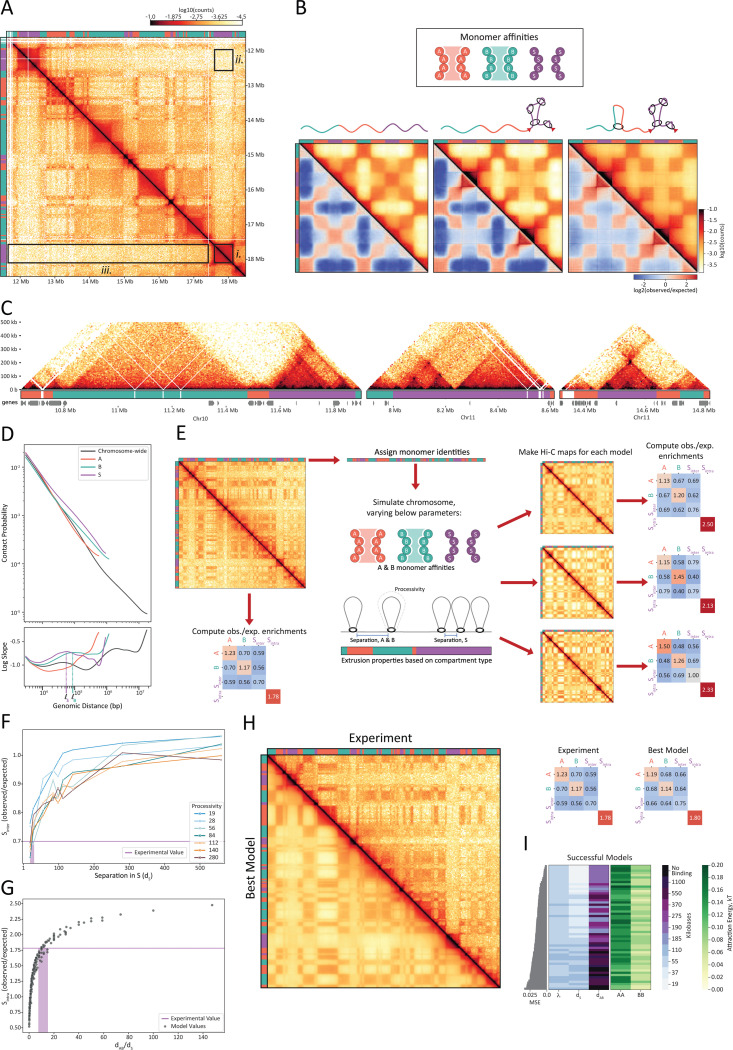
Simulations of affinity-based compartmentalization and activity-based loop extrusion recapitulate Hi-C contact patterns. (**A**) Hi-C map and compartment annotations highlighting the three distinct features of S compartments. Data correspond to Chr22: 11,625,000–18,435,000 from PD-D2 sample. (**B**) Minimal models of A, B, and S compartmentalization with A-A and B-B affinities (both set to 0.075 kT) and S-S and heterotypic interactions set to 0.00 kT. *In silico* Hi-C and observed-over-expected Hi-C maps for models of (*left*) compartments only; (*middle*) compartments plus high extrusion S; (*right*) compartments plus high extrusion in S and low extrusion in A and B. (**C**) Evidence of loop extrusion in the PD-D2 Hi-C data including insulated domains, stripes, and corner peaks. Three regions of the PD-D2 Hi-C maps at 5 kb resolution, with compartment and gene tracks below. The regions correspond to (*left*) Chr10: 10,640,000–11,900,000; (*middle*) Chr11c: 7,900,000–8,615,000; (*right*) Chr11c: 14,310,000–14,820,000. (**D**) (*Top*) The contact probability P(s) curve for PD-D2 Hi-C as a function of genomic distance (s). (*Bottom*) Log-derivative of contact probability as a function of genomic distance. Curves represent averages of either chromosome-wide (gray) or for contiguous segments of a given compartment type (colored). Estimates of the average loop size in S (*ℓ*_S_) and in B (*ℓ*_B_) are noted along the x-axis. (**E**) General workflow for comparing the experimentally generated Hi-C to the polymer models of Chr15: 0–6,500,000. (**F**) Observed-over-expected values of S-S_inter_ plotted as a function of separation between loop extruders in S (d_S_) for a series of polymer models. Each curve represents a series of models, which share loop extruder processivity. All models lacked extrusion in A and B chromatin and shared homotypic attractions of 0.12, 0.04, and 0.00 kT for A, B, and S, respectively, to yield an A/B compartment strength similar to the experimentally generated Hi-C. The experimental value of S-S_inter_ is shown as a purple line. (**G**) Observed-over-expected values of S_intra_ plotted as a function of relative abundance of loop extruders (d_AB_/d_S_) for a series of polymer models, each with different processivities and separations. The experimental value of S_intra_ is shown as a purple line. (**H**) Best polymer model versus the experimental data for Chr15: 0–6,500,000. The best model’s parameters are A-A attraction energy = 0.16 kT, B-B attraction energy = 0.08 kT, S-S attraction energy = 0.00 kT, λ = 55 kb, d_S_ = 19 kb, d_AB_ = 190 kb. (*Left*) Experimentally generated Hi-C (*top, right half of the map*) versus the *in silico* generated Hi-C map from the best model (*bottom, left half of the map*). (*Right*) Summary statistics (observed-over-expected off-diagonal compartment and on diagonal S_intra_ enrichments) for the experimentally generated data (*left*) and best model (*right*). (**I**) Summary of successful polymer models. Parameter values for (*center*) extruder properties and (*right*) homotypic A-A and B-B affinities. Models were sorted by (*left*) the mean square error of their observed-over-expected summary statistics versus those of the experimental data (top-most parameter set corresponds to the best model visualized in Figure 4H).

Given the novel and stark contact pattern of compartment S (Figure 4A), we searched for a possible mechanistic model underlying its formation. To reproduce this pattern, we generated a series of polymer models with alternating, equal-sized A, B, and S domains. For each model, we simulated an ensemble of equilibrium conformations from which we generated *in silico* Hi-C maps (Figure 4B and M&M). We selected models based on their ability to reproduce the following three Hi-C features that together make S domains unique (Figure 4A): (*i*) contacts are enriched within contiguous S domains (locally enriched, S_intra_); (*ii*) each S domain is depleted in compartmental contacts with other S domains (distally depleted, S-S); (*iii*) the depletion of contacts between S domains and all compartment types is homogenous (smooth lanes of depletion, S-S ≈ S-A ≈ S-B).

Given the clear compartment patterning of A and B regions in the Hi-C, we first sought to explain the overall patterning via a simple three-compartment system with homotypic affinities (*i.e.*, applying A-A, B-B, and S-S attractions) while keeping heterotypic attractions neutral and all-monomer repulsion (Figure 4B, left). Though we systematically explored the parameter space for homotypic affinities (Figure S5A), we found that none of the models satisfied our criteria. While we could reproduce conventional checkerboard patterning as desired for A and B compartments, we could not reproduce the features of S. Models with S-S affinity performed even worse than those in which S attractions were kept neutral. We therefore concluded that compartment S cannot result from affinity-mediated compartmentalization alone.

To explain the missing S features in the compartment-only models, we turned to loop extrusion. In vertebrates, the SMC complex cohesin extrudes loops and, when occluded by extrusion barriers, generates domains of local contact enrichment known as TADs. Another effect of loop extrusion is a decrease in compartmentalization ^14,56,57^. We therefore hypothesized that loop extrusion could be sufficient to resolve the three missing S features in our models. To test this, we devised a model of loop extrusion exclusively within S regions, with the ends of S domains acting as barriers to extrusion. Indeed, when applied to a model with A and B homotypic affinities, extrusion successfully enriched local S contacts (feature *i*) and depleted distal contacts between pairs of S domains (feature *ii*). However, S-specific extrusion did not generate smooth lanes of depletion (feature *iii*) (Figure 4B, center). We loosened our assumption of extrusion occurring exclusively in S by introducing some level of loop extrusion to non-S regions. By varying the levels of extrusion outside of S, we found that only models with low levels of extrusion in A and B relative to S were successful (Figure 4B, right, M&M). As the degree of extrusion in A and B approached that of S, distal S-S enrichment re-emerged (loss of feature *ii*) (Figure S5B).

With the working model of A and B self-affinities and S-rich extrusion, we revisited whether homotypic S attraction inhibited the formation of S features. We found that introducing homotypic attraction to S (comparable to that of A-A and B-B) does not alter Hi-C maps (Figure S5C). Thus, S-like features can be produced with or without some level of S-S attraction, so long as loop extrusion in S is sufficiently high to counteract the effects of such affinity.

To generate the unique A, B, and S patterning, we converge on a class of models where chromosome organization is established by two mechanisms: A and B homotypic attractions driving compartmentalization; and basal levels of extrusion along the chromosome with higher levels of extrusion in S, thereby limiting its contact with the rest of the chromosome.

### Evidence of loop extrusion in B. mori Hi-C maps

To evaluate whether extrusion as predicted from our models was supported by the data, we searched for evidence of loop extrusion in the PD-D2 Hi-C data. Key indicators of loop extrusion activity are the presence of specific Hi-C patterns such as insulated domains, dots, and stripes that also require extrusion barriers ^58^, and the shape of the contact frequency *P(s)* as a function of genomic separation *s* ^59,60^. While the resolution of compartment annotations limits our ability to systematically detect individual dots, stripes, and insulation points for all compartment types and sizes, we found many examples of barrier-restricted extrusion throughout the genome. We frequently found such features nested within each other, inside, and at the edges of many S domains (Figure 4C). Furthermore, in the genome-wide *P(s)* curve (Figure 4D) computed from the PD-D2 Hi-C maps, we indeed observe an extrusion-indicative shoulder and a corresponding peak in the log-derivative of the *P(s)* curve. The peak in the log derivative plot represents the average loop size ^59,60^, which in this case is approximately 40–60 kb. We also computed these curves separately for continuous segments of each compartment type (Figure 4D). Consistent with the proposed mechanism, the P(s) shoulder is most prominent for compartment S and likewise indicates an average loop size of 40–60 kb. Although less pronounced, the curve corresponding to compartment B also indicates loop extrusion activity, though with more sparse loops, as suggested by the smaller height and right-shift of the peak.

We find strong evidence that loop extrusion occurs across interphase chromosomes in *B. mori*. Consistent with our phenomenological model, the Hi-C data also suggests that loop extrusion is non-homogenously distributed, with extruders showing preference for S domains.

### Integrated model of a B. mori chromosome using affinity-based compartmentalization and S-enriched loop extrusion

To understand how the interplay of loop extrusion and compartmentalization shapes the *B. mori* genome, we developed a quantitative chromosome-scale model employing the principles learned from the minimal model and measurements uncovered from the Hi-C data (Figure 4E). We sought to recapitulate the first 6.5 Mb segment of Chr15 in a polymer model. To best capture the strong territoriality of *B. mori* chromosomes, we simulated this segment in spherical confinement. We assigned compartment identities based on those obtained from PD-D2 Hi-C data and introduced self-affinities to A and B accordingly. We tested models with and without extrusion. Altogether, the models had five parameters that we investigated: A-A affinity, B-B affinity, extruder processivity, the average separation between extruders inside S (d_S_), and the average separation between extruders outside of S (i.e., inside A&B regions, d_A&B_). The processivity (λ) is defined as the average size of a loop extruded by an unobstructed extruder (no collisions with barriers or other extruders). Assuming that the same motor extrudes loops across the genome, we applied the same processivity of extruders across the full region, regardless of the underlying compartment type.

We first simulated our chromosome models by varying A and B homotypic affinities in the absence of loop extrusion. In doing so, we reaffirmed a key finding of the phenomenological model: compartmentalization of A and B alone cannot create secluded S domains (Figure S6A). By introducing extrusion to our models, we found that different aspects of this process control different features of S. Interestingly, we found that S-to-S compartment depletions (feature *ii*) were primarily driven by the separation between extruders in S (d_S_) (Figure 4F) rather than extruder processivity (Figure S6B). To generate distal S depletions similar to those measured in the Hi-C, the abundance of extruders in S must be high (separations must be small). Based on our selected region of Chr15, where the observed-over-expected S_inter_ (feature *ii*) depletion was 0.7, we estimate the separation d_S_≈20–40 kb (Figure 4F).

The ratio of processivity to separation (λ/d) controls chromatin compaction, with higher values yielding higher degrees of compaction ^61^. We found that even low λ/d_S_ ≈ 1 can achieve S-to-S compartment depletions (feature *ii*), which indicates that severe compaction itself is not necessary to seclude S from the rest of the chromosome. Moreover, models with compact, mitotic-like extrusion (λ/d_S_ ≈ 10) yielded overly pronounced shoulders in their P(s) curves, inconsistent with Hi-C data (Figure S6C).

We found that we could determine the separation of extruders in A and B (d_A&B_) based on the compaction of S (S_intra_) relative to A and B and our estimate of separation in S (d_S_). To generate the desired degree of relative compaction in our models (S_intra_ = 1.78, feature *i*), we estimate that extruders are 8- to 15-fold more abundant in S compared to A and B (i.e., d_A&B_/d_S_≈8–15, Figure 4G). From the estimated loop extruder density in S (d_S_≈20–40 kb) and the average loop size of 40–60 kb inferred from Hi-C, we chose the highest and lowest processivities that yielded the average loop size in this desired range (λ = 55 and 110 kb) (Figure S6D).

The degree of A/B compartmentalization can modulate the effects of extrusion on our measured features and vice versa (Figures S6E and S6F). We therefore widened the estimated ranges for extrusion parameters (d_S_, d_A&B_, and λ) and simulated all combinations of these parameters with varied monomer affinities. After testing 1,710 models, 62 (3.6%) recapitulated all three S criteria and A/B compartmentalization. We identified shared features among these models, as they may underlie the components key to folding the *B. mori* genome. First, we found that attraction energies among A-type monomers are consistently greater than those of B-type monomers, often by a factor of approximately two (Figure 4I, *left*). This suggests that A-A attractions play a key role in the compartmentalization of *B. mori* chromatin, which is in contrast to studied mammalian genomes, where compartmentalization is driven largely by B-B interactions ^62^. Second, we found that S smoothness (feature *iii*) was dependent on the degree of A/B compartmentalization (r = 0.74, p<10^−8^), and all models with realistic S smoothness had relatively weak compartmentalization of both A and B chromatin (Figure S6G). Third, we found that the best models were enriched with relatively lower looping densities within S (λ/d_S_ ≈ 0.5–2), indicating that, in S, the density of loops is similar to estimates of interphase vertebrate chromatin ^13,63^. Finally, nearly all successful models (97%) contained some degree of extrusion in A and B chromatin (Figure 4I, *right*). Lower densities of extruders in A and B outperformed those with higher densities (d_A&B_/d_S_ = 10 was enriched over d_A&B_/d_S_ = 5 or no A&B-binding).

Our models reveal previously uncharacterized behaviors for loop extrusion in shaping the genome. In the case of *B. mori*, the models suggest that loop extrusion is localized primarily within compartment S, leading to its higher compaction and relative seclusion. Parameter estimation suggests that loop sizes and extrusion density within S are comparable to those in the mammalian interphase. Our models show that the interplay between euchromatin-driven compartmentalization and non-uniform extrusion explains the unique folding of the *B. mori* genome.

### Compartment *S is found preferentially on the surfaces of chromosome territories*

**Figure 5:**
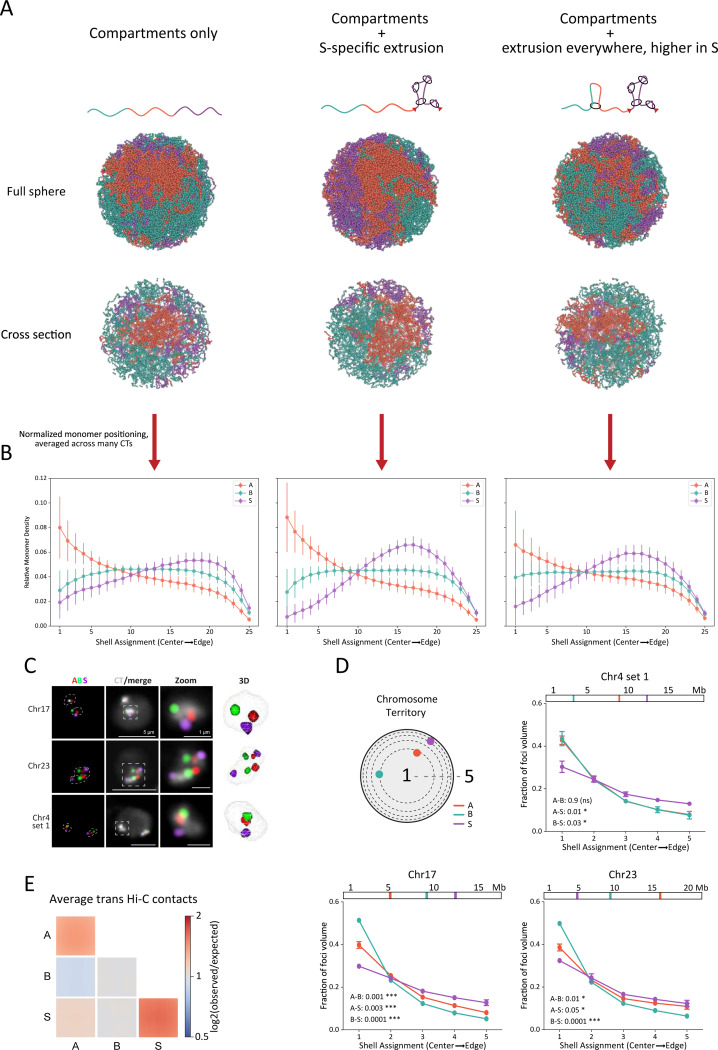
Compartment S is peripherally located within chromosome territories, with modeling indicating a role for loop extrusion in this positioning. (**A**) Example conformations for three models of Chr15, with the top row of renderings reflecting the entire sphere and the bottom row of renderings representing cross-sections through the center of the sphere. Each column represents a model introducing a new source of organization to the chromosome; (*left*) a rendering of a compartment-only model (A-A attraction energy = 0.16 kT, B-B attraction energy = 0.08 kT, S-S attraction energy = 0.00 kT); (*middle*) a rendering of the same compartment model with loop extrusion within S only (λ = 55 kb, d_S_ = 19 kb); (*right*) a rendering of the same compartment model as the far left but with different loop extruder densities in S versus A and B (λ = 55 kb, d_S_ = 19 kb, d_AB_ = 190 kb; the best model from the previous section). (**B**) Relative monomer densities for A, B, and S monomers for the three models detailed in Panel A. Dots represent mean values, while lines represent the standard deviation. (**C**) 4-color Oligopaint FISH labeling single A (red), B (green), and S (purple) domains as well as the corresponding CT (white). First column: Oligopaint labeling of domains, with white dashed lines indicating CT edges. Second column: Oligopaint labeling of domains merged with Oligopaint labeling of CTs. Third column: zoom-in views corresponding to boxes traced in column two. Fourth column: 3D rendering of zoomed CT from TANGO ^91^. Microscope images are Z-projections of 10 Z stacks. The background in the CT channel acts as a proxy for the nuclear edge. (**D**) Shell analysis measuring foci positions within their CTs for Chr04, 17, and 23. The location of Oligopaint FISH probes within the chromosome is shown above each plot. Dots indicate means of 3 biological replicates (different embryos, n>250 nuclei). Error bars show standard error of mean. P-values were generated from unpaired t-test (Kolmogorov-Smirnov test) between distributions and are indicated at the bottom left of each graph. (**E**) Average observed-over-expected *trans* contacts within and between all A, B, and S compartments.

Compartmentalization drives the spatial partitioning of active and inactive chromatin within the nucleus ^62^. Using our best model of Chr15, we asked whether the “sequestered” compartment S displays distinct spatial positioning. By analyzing the radial positioning of A, B, and S, we found a strong preference for S domains to be at the periphery of CTs (Figures 5A, 5B). A and B both showed preference for localizing to the core of the territory, with A being more central. When we compared this to an analogous model without extrusion, S was more interspersed throughout the territory as a whole. This reduced preference toward the exterior of the territory indicates that extrusion in S drives its peripheral localization.

To follow up on this result, we used Oligopaint FISH to label portions of single A, B, or S domains as well as the whole CTs for Chr04, Chr17, and Chr23 in embryonic nuclei (Figures 5C and S7). Shell analysis (see M&M for details) revealed that S domains are more likely to occupy peripheral CT shells compared to A and B domains (Figure 5D). In addition, measuring the distance from the domain center to the CT edge also showed that S domains are closer to the CT edge than A or B domains for all chromosomes and loci analyzed (Figure S7).

Although inter-chromosomal contacts were sparse in the Hi-C data, we asked whether this peripheral localization could influence the average contact frequency among S domains in *trans*. Therefore, we computed the average *trans* observed-over-expected contacts between each compartment type (Figure 5E). We found that the average value of S-S *trans* contacts is higher compared to any other combination in *trans*, and, in particular, A-A and B-B. This is consistent with the preferential positioning of S towards the periphery of CTs, a favored location for *trans* contacts.

Our DNA FISH reveal S domains are preferentially located at the CT peripheries, which is further supported by the Hi-C. Our models indicate that this may be caused by a previously unknown effect of loop extrusion: its ability to influence the spatial positioning of chromatin in the context of its chromosome territory.

### Compartment S genomic localization changes during development

**Figure 6:**
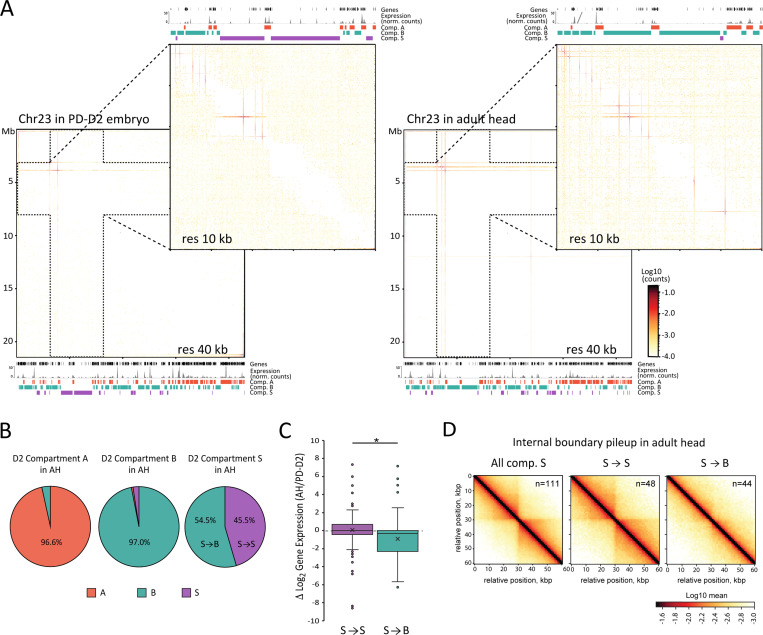
Compartment S changes during development. (**A**) Hi-C contact maps of the full Chr23 at 40 kb resolution and Chr23:3,000,000–80,000,00 region at 10 kb resolution in PD-D2 embryos on the left and in adult head (AH) on the right. Below or above each matrix are gene locations, RNA-seq expression aggregated at 1 kb resolution and compartments A, B, and S genomic location, for the corresponding stage and region. (**B**) Compartment assignment in AH datasets of large domains (>200 kb) assigned to large compartment A, B or S in PD-D2, in % of the compartments assigned in PD-D2. (**C**) Box plots of distributions of log_2_ ratio of gene expression (TPM) between PD-D2 embryos and AH. In purple is the distribution for genes that are in domains assigned to compartment S in the two stages (S→S) (n=119), and in green is the distribution for genes that are in region assigned to compartment S in PD-D2 and that switched to B in AH (S→B) (n=120). Boxed region corresponds to data between first to third quartile. Line indicates median of distribution while cross corresponds to mean. Whiskers extend to lowest and highest data points, and dots outside correspond to outliers. Asterisks indicate distributions significantly different by Kolmogorov-Smirnov test (*: P-value = 0.01 – 0.05). (**D**) Pileup plots centered on internal boundaries within all large S compartment domains (>200 kb, excluding 40 kb on each side of domain boundaries) in AH or S→S and S→B categories, as described in the right panel of (B), at 5 kb resolution and extending to 30 kb on each side.

Taking advantage of our Hi-C datasets from different developmental stages, including three embryonic stages and one adult stage, we next explored the developmental dynamics of compartment S. Based on initial visual inspections, we could identify domains switching to or from S on multiple chromosomes between timepoints (see examples in Figure S8A). To test the dynamics of S compartment switching more systematically, we repeated the compartment calling protocol for the adult stage (Adult Heads, AH). We found that a comparable fraction of the AH genome (12%) folds into compartment S, compared to the PD-D2 embryonic stage (Figure S8B). Nevertheless, several embryonic S domains visually show checkerboard patterning in the AH Hi-C maps, including three of the largest S domains on Chr06 and Chr23 (Figures 6A and S8C). To compare the two datasets further, we restricted our analyses to large (>200 kb) domains to allow for visual confirmation of compartment assignment based on the Hi-C contact pattern. We found that only about 45% of S domains defined in the PD-D2 embryonic stage are maintained in the AH (referred to as S->S) (Figure 6B). This fraction is much lower compared to that of the A or B domains. Large S domains that change compartment assignment in the AH exclusively turn into B domains (referred to as S->B). Notably, this change coincides with a significant reduction in gene expression levels (Figures 6C and S8D). The Hi-C contact maps at S domains that turn into B show a loss of features associated with loop extrusion, including insulation points and off-diagonal dots (Figures 6A and S8C). Weakening of insulation is also evident when comparing pileup contact enrichments of boundaries called within S domains that are maintained (S->S) or lost (S->B) in the AH data (Figure 6D). The loss of extrusion features and intra-domain compaction, together with the increased checkerboard patterning of S domains that turn into B, are consistent with our model that loop extrusion underlie the spatial segregation of compartment S by counteracting compartmentalization. Finally, the developmental dynamics of several S domains also argue against a strict genetic specification of compartment S but rather support the presence of epigenetic features involved in its formation.

## Discussion

Our investigation of *B. mori*’s genome organization reveals both conserved principles and novel folding behaviors. As seen in other eukaryotes, we observe the formation of strong CTs and spatial segregation of chromatin into active A and inactive B compartments. Unlike other eukaryotes, we have observed much stronger chromosome territoriality and a novel type of compartment, which lacks the characteristic checkerboarding of A/B compartments and appears to be rich in loop extrusion activity.

The remarkably strong CTs and the low frequency of inter-chromosomal contacts are consistent with a recent whole chromosome Oligopaint study of six *B. mori* chromosomes, which likewise revealed highly spatially distinct CTs ^44^. In the context of a recent study categorizing a variety of eukaryotic genomes into two types of architectures, Rabl-like (I) and strong CT (II) ^3^, *B. mori* represents an extreme case of type II. In that study, as well as a study in *Drosophila melanogaster*, folding, volume, and intermixing of CTs have been associated with condensin II ^3,64^. Condensin II subunits are present in the *B. mori* genome ^65,66^, and it would be interesting to evaluate whether the substantial degree of territoriality is caused by uniquely high activity of condensin II in *B. mori*. CT strength is also intriguing from an evolutionary point of view. Previous studies in *D. melanogaster* cell lines ^67^ and across human cancers ^68^ have described an inverse relationship between the frequency of inter-chromosomal contacts and the incidence of genomic translocations. Consistent with these studies, karyotypes and synteny are highly conserved across Lepidoptera, including *B. mori* ^69^, suggesting that the strong CTs in *B. mori* may contribute to low structural variations and high karyotype conservation in these organisms.

The presence of compartment S, with domains that strongly self-interact but segregate away from the rest of their chromosome, is remarkable. To our knowledge, there is no precedent for a compartment with similar contact or epigenetic profiles as compartment S. While other compartment types beyond A and B have been detected ^2,34,70^, they typically represent sub-types of A and B and show preferential contacts with domains of the same type ^34^. Although the previously described “intermediate” compartment I ^14,71,72^ shares some characteristics with compartment S, such as H3K27me3 enrichment and developmental plasticity, they differ in their most prominent features. Compartment I is enriched in contacts with A, B, and I. A recently identified sub-compartment in HCT116 cells, termed B_0_^34^, likewise displays relatively low levels of compartment contrast (i.e., its compartments are smoother and checkerboard less than other compartment types). Domains of compartment S, on the contrary to I or B_0_, are homogenously *depleted* in contacts with any other domain. Furthermore, compartment S is the only compartment type to display high levels of contacts within contiguous domains, despite the lack of preferential contacts between pairs of domains. This pattern, plus their gene composition and distinct epigenetic makeup, make S unique compared to any previously identified compartment.

The formation of compartment S likely requires a mechanism distinct from any other described organism. While conventional compartmentalization is believed to rely on affinity between regions that share epigenetic composition ^73^, we propose that compartment S is formed via localized loop extrusion activity. Not only does this loop extrusion activity lead to the formation of dense and secluded S domains, but it also drives their peripheral localization within CTs. This effect of loop extrusion is a novel finding, raising the possibility that loop extrusion may similarly affect large-scale organization in other organisms or contexts. The underlying physics by which extrusion is capable of achieving these structures is yet to be understood.

Importantly, in vertebrates, loop-extruding cohesin (during interphase) and condensins (in metaphase) are generally believed to load uniformly across the genome, thereby showing no preference for a specific compartment type ^34,74^. In our models, the formation of compartment S in *B. mori* requires higher densities of loop extruders within S relative to A and B. Such localization of extruders to many broad domains (tens to hundreds of kb each) has not been identified in other systems and would require targeted loading of loop extruding factors in compartment S. While historically proposed targeted loading at CTCFs or transcription start sites has been found inconsistent with new data ^75,76^, other sites and mechanisms of targeted loading are being discovered. Targeted SMC loading to certain genetic elements is well-described across various biological systems. In the bacterium *B. subtilis*, condensins are loaded at ParS sites by the ParB DNA binding protein ^77,78^. In yeast, it has been suggested that sequence context antagonizes SMCs from centromere binding ^79^. In *C. elegans*, specific sequences direct the SMC-containing dosage compensation complex to ^36,80^. Most recently, targeted loading of SMCs has been reported at enhancers in *C. elegans* ^11,81^ and in vertebrates ^10,12,82^. Such targeted loading in these systems may be guided by differences in DNA accessibility, sequence-specific DNA binding proteins ^77,78^, or via specific histone marks ^10,12,82^.

The presence of loop extrusion domains in *B. mori* is supported by simulations, and importantly, by characteristic patterns in Hi-C maps. The presence of dots, stripes, and nested domains with insulation indicate the presence of extrusion barriers, similar to CTCF in vertebrate systems^75^, RNA and DNA polymerases^76,83^, and MCM complexes ^84^. This raises the possibility of extrusion barriers such as CTCF, CP190, and Mod(mdg4), which are conserved in *B. mori* ^85,86^.

In the context of targeted loading of SMC complexes, the enrichment of H4K20me1 in compartment S is interesting. In *C. elegans*, it has been shown that the SMC-based dosage compensation complex enriches H4K20me1 on the inactive sex chromosome by means of a demethylase ^52^. H4K20me1 has deposited during the process of silencing and compacting the X chromosome in mice ^87^. Compartment S shares similarity to inactivated sex chromosomes by virtue of being locally compacted and isolated from the rest of the genome, while it is distinct from inactivated sex chromosomes by virtue of its permissiveness to gene expression. Whether enrichment of H4K20me1 aids in chromatin compaction, and what its relationship is to loop extrusion, is unclear for *B. mori*.

The function of compartment S and the role of loop extrusion there remain intriguing questions. First, in view of the holocentric architecture of *B. mori* chromosomes, we consider the possibility that compartment S is involved in centromere specification to be unlikely. This conclusion is guided by our finding that the genomic distribution of S domains (Figure S3C) does not correlate with centromere profiles that we generated from a *B. mori*-derived cell line ^88^. Instead, the isolated genomic environment of S domains might ensure the precise transcriptional regulation of the genes that they contain. Our finding that S-located genes are functionally enriched in transcription-related processes might suggest that S represents a developmental transition state to either A or B, as hypothesized for compartments I and B_0_^34,71,72^. Such a model is supported by the observation that many S domains are variable among different developmental stages. Furthermore, S domains may represent development-control units such as the Hox cluster, which comprises a large S domain in *B. mori* embryos (Figure S4). By analogy to the Hox cluster, where loop extrusion appears to be key to the precise sequence of gene activation^89^, compartment S may recruit a high density of extruders to achieve precisely timed activation of genes during development. Our observation of developmental plasticity of S domains further supports this hypothesis.

Broadly, our observation of localized loop extrusion in *B. mori* may also hold true for other insects. While some studies resolve conflicting evidence of this process in another insect, *D. melanogaster*, where some signatures of extrusion are evident in Hi-C but other signatures are missing ^90^. It is possible that, akin to *B. mori*, *D. melanogaster* chromatin has loop extrusion activity localized to specific genomic regions.

In summary, our study describes the unique organization of the *B. mori* genome, focusing on its exceptional degree of chromosome territoriality and the discovery of a new genome folding structure. We propose that this novel structure, compartment S, is formed by loop extrusion localized to a specific compartment type. This work both expands our knowledge of the possible structures of genomes and also provides novel insights into the interplay of two major processes governing genome folding: loop extrusion and compartmentalization. Our work highlights how the diversity and plasticity of genome organization can arise from this interplay. We thus demonstrate the power of researching non-model organisms in the field of genome organization.

## Figures and Tables

**Table 1:** ShinyGO Molecular Function enrichment of *B.mori* genes in S.

Pathway	GO ID	Enrichment FDR	Number of genes in category	Total number of pathway genes	Fold Enrichment

DNA binding	GO:0003677	6.10E-23	66	592	4.45
Nucleic acid binding	GO:0003676	7.43E-14	89	1461	2.43
DNA-binding transcription factor activity	GO:0003700	3.18E-13	31	220	5.63
Transcription regulator activity	GO:0140110	5.13E-12	32	262	4.88
Sequence-specific DNA binding	GO:0043565	7.10E-08	19	139	5.46
DNA-binding transcription factor activity, RNA polymerase II-specific	GO:0000981	2.811E-06	13	80	6.49
Nuclear receptor activity	GO:0004879	4.05E-03	4	12	13.32
Ligand-activated transcription factor activity	GO:0098531	4.05E-03	4	12	13.32
Steroid hormone receptor activity	GO:0003707	1.22E-02	4	16	9.99
Signaling receptor binding	GO:0005102	2.77E-02	7	67	4.17
